# Work-related thumb disorders in South African physiotherapists treating musculoskeletal conditions using manual therapy techniques

**DOI:** 10.4102/sajp.v71i1.249

**Published:** 2015-05-29

**Authors:** Heather Jenkins, Hellen Myezwa

**Affiliations:** 1 Department of Physiotherapy, University of Witwatersrand, South Africa

## Abstract

**Research question:**

What is the prevalence of and factors associated with work-related thumb problems (WRTP) in South African physiotherapists treating musculoskeletal conditions using manual therapy techniques?

**Design:**

A cross-sectional, descriptive study design was used and data were collected using two Internet-based questionnaires.

**Participants:**

The sample size calculated for the study was 284 using 95% confidence levels and a 5% margin of error. There were 395 participants that were included in the study.

**Outcome measures:**

The variables measured included demographic, employment, educational and occupational factors.

**Results:**

The lifetime prevalence of WRTP in the physiotherapists was 65.3%. The manual techniques that were significantly associated with WRTP in the respondents who reported thumb problems were all grades of transverse glides applied to the spine as well as grade II–IV unilateral and central posterior-anterior pressures to the spine. The factors that remained significantly associated with WRTP in all 395 respondents after regression analysis were the cervical treatment of up to six patients a day and hyperextension > 30° of the non-dominant interphalangeal (IP) joint of the thumb.

**Conclusion:**

This study confirms that a high percentage of physiotherapists using manual therapy techniques to treat musculoskeletal conditions are experiencing WRTP.

**Recommendations:**

The development of a valid and reliable WRTP screening tool is needed to aid in the identification of physiotherapists at risk and thus in the primary prevention of WRTP. A longitudinal study which follows newly qualified physiotherapists is recommended to investigate a possible cause-effect relationship and preventative strategies for WRTP in physiotherapists.

## Introduction

The World Health Organization (Luttman, Griefahn & Caffier 2003) defines a work-related musculoskeletal disorder (WRMSD) as a form of ill health ranging from light transitory disorders to irreversible disabling injuries that are induced or aggravated by work and related circumstances. Despite physiotherapists’ specialist knowledge on injury prevention, they are also prone to WRMSD. Several international studies (Bork *et al.* 1996; Holder *et al.* 1999) reported a 32% lifetime prevalence of WRMSD in physiotherapists, whilst Cromie, Robertson and Best (2000) and West and Gardner (2001) reported a higher lifetime prevalence of WRMSD of 91% and 55% respectively. African studies (Obembe *et al.* 2008; Useh, Igumbor & Madzivire 2001) reported a lifetime prevalence of 78% whilst another study (Adegoke, Akodu & Oyeyemi 2008) reported an annual prevalence of 91%.

A hands-on approach of manual therapy is predominantly used in orthopaedic outpatient departments or private practices treating musculoskeletal disorders. Increased use of their hands puts these physiotherapists at risk of developing WRMSD of the wrists and hands (Barnes *et al.* 2011; Cromie *et al.* 2000; McMahon, Stiller & Trott 2006; Snodgrass & Rivett 2002; West & Gardner 2001). The lifetime prevalence of work-related thumb problems (WRTP) ranged from 62.5% to 83% in studies focusing on wrist and thumb disorders (Barnes *et al.* 2011; McMahon *et al.* 2006; Wajon & Ada 2003).

Barnes *et al.* (2011) investigated the prevalence of work-related wrist and thumb pain and the contributing risk factors amongst physiotherapists working in Bloemfontein, South Africa. The current study reported on WRTP only and included a national sample of physiotherapists.

## Methodology

### Design

An observational study design was undertaken using a cross-sectional, Internet-based questionnaire.

### Sample

#### Sample size

A sample size was calculated using the formula sample size = *t*² × *p* (1–*p*)/m², where *t* = 1.96 for a 95% confidence level, m = margin of error at 5% (standard deviation of 0.05) and *p* = 0.65 for a 65% prevalence of WRTP in a similar national study of WRTP in physiotherapists (McMahon *et al.* 2006). The sample size calculated (International Fund for Agricultural Development n.d.) was 350.

#### Inclusion criteria

South African physiotherapists registered with the South African Society of Physiotherapists (SASP) who were treating or have previously treated musculoskeletal conditions using manual therapy techniques were included in the study.

#### Exclusion criteria

Physiotherapists with structural deformities or injuries to the upper limbs as a result of non-work-related causes as well as those with diseases affecting the hands were excluded.

### Outcome measures

A questionnaire was developed using information from similar studies (Barnes *et al.* 2011; McMahon *et al.* 2006; Wajon & Ada 2003). It comprised demographic information, general questions on the area of practice; years worked as a physiotherapist; number of years or patients treated in the different body regions; and whether preventative education was received. Specific questions about WRTP regarding the thumb affected, symptoms, history of onset, aggravating factors and management strategies were included. The information on gender was elicited from a second questionnaire as it was excluded from the initial questionnaire because of a technical error. The questionnaire was validated for content by a panel of eight ‘experts’, who included physiotherapy lecturers and clinical physiotherapists. Further piloting of the questionnaire on 12 physiotherapists (questionnaires not included in the main study) was done to enhance the content validity of the questionnaire. The question in which the physiotherapists had to quantify the time spent on manual therapy was removed because of difficulty in quantifying time spent on manual techniques. McMahon *et al.* (2006) did not find a significant association between time spent and WRTP amongst 1562 physiotherapists of whom 65% had WRTP.

### Ethical considerations

Ethical clearance was obtained from the Human Research Ethics Committee at the University of the Witwatersrand (number M120429). All SASP-registered physiotherapists throughout South Africa were sent an Internet link to the survey and asked to complete the survey. A reminder was sent twice in the collection period between August 2012 and February 2013. In addition, physiotherapists belonging to the Orthopaedic Manipulative Therapy Group and the Sports Group received a link from their special interest group secretary which served as an additional reminder. Confidentiality was insured as the questionnaire did not request names, identity numbers and email addresses.

### Data analysis

Data collected from the online questionnaire were exported to Microsoft Excel 2010 and then to Stata/IC 10.0 software to be analysed. Descriptive analysis was used to reduce the categorical data to frequencies and percentages. Tests for association were conducted using the Pearson's chi squared test for categorical data. Relationship of the data that were significantly associated with WRTP in all 395 respondents was tested in a regression analysis. Testing was done at the 0.05 level of significance.

## Results

Of the 3523 physiotherapists who received the link, 456 responded to the invitation to participate in the study. Of these, 61 were excluded from the study on the grounds of missing data (27), structural deformities (21), diseases of the hand (8) and operations to the forearm and hand as a result of non-work-related factors (5), resulting in a final sample size of 395. This represented 13% (*n* = 45) more respondents than the calculated (*N* = 350) sample size.

The demographics of the sample are presented in Table 1. The majority of the respondents who responded to the questionnaire on gender (60%, *n* = 243) were female (93%, *n* = 226). The lifetime prevalence of WRTP in the current study was 65.3% (*n* = 258). The greatest majority respondents with WRTP was found in the age group 20–30 years (70%, *n* = 84).

**TABLE 1:** Demographic characteristics including prevalence of work-related thumb problems.

Demographic characteristic	Specification	Total respondents	%	Respondents with WRTP	%
Age in years	20–30	120	30.4	84	70.0
	31–40	127	32.1	78	61.4
	41–50	88	22.3	55	62.5
	51–60	45	11.4	3	73.3
	> 60	15	3.8	8	53.3
Height in cm	< 150	4	1	3	75.0
	151–160	83	21	56	67.5
	161–170	164	41.5	108	65.8
	171–180	122	30.9	76	62.3
	> 180	22	5.6	15	68.2
Weight in kg	< 50	16	4.0	13	81.2
	50–60	129	32.6	85	65.9
	61–70	118	29.9	73	61.9
	71–80	74	18.7	52	70.3
	81–90	39	9.9	23	59.0
	> 90	19	4.8	12	63.1
Race	Asian	20	5.1	14	70.0
	Black	12	3.0	6	50.0
	Mixed	27	6.8	16	59.3
	White	336	85	222	66.1
Qualifications	OMPTG	199	50.4	130	65.3
	Nil	141	35.7	92	65.2
	Other	48	12.1	9	18.7
	Sports and Exercise	35	8.9	18	51.4
	Community Health	13	3.3	9	69.7
	Paediatrics	8	2.0	6	75.0
	Neurology/Neurosurgery	6	1.5	5	83.3
	Respirology/Cardiothoracic	4	1.0	3	75.0
	Orthopaedic Surgery	2	0.5	2	100
	Trauma	2	0.5	2	100

WRTP, work-related thumb problems; OMPTG, Orthopaedic Manipulative Physiotherapy Group.

**FIGURE 1:**
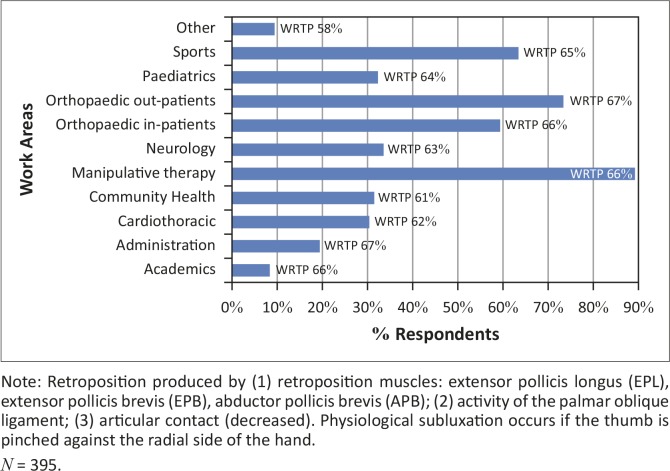
Work areas and prevalence of work-related thumb problems in respondents.

Most respondents were employed full time at the time of the survey (89.1%, *n* = 352). The majority of respondents worked in work areas using manipulative therapy (Figure 1). The greatest prevalence of WRTP was found in physiotherapists working with orthopaedic outpatients and in work areas using manipulative therapy. Preventative education was only received by 47.1% (*n* = 186) of the respondents. The majority of respondents received education on thumb protection at undergraduate level (28.1%, *n* = 111).

Table 2 represents the self-reported generalised hypermobility of respondents. Although a minority of physiotherapists reported generalised hypermobility for four out of the five parameters (< 15.4%, *n* < 61), the majority of the respondents (> 44%, *n* = 174) reported hypermobility of the joints of the thumbs between 0° and 30°, as shown in Table 3. The highest prevalence of WRTP was found in respondents with interphalangeal (IP) joint hyperextension greater than 30° (dominant: 75.6%, *n* = 62; non-dominant: 78.5%, *n* = 62), as shown in Table 4.

**TABLE 2:** Distribution of generalised hypermobility and association with work-related thumb problems.

Generalised hypermobility	Category	Total	%	p-value
Elbow hyperextension > 10°	Yes: Dominant	59	15.0	0.9
	Yes: Non-dominant	46	11.6	0.5
	No	334	84.5	-
	**Total**	**439**	**111.1**	**-**
Knee hyperextension > 10°	Yes: Dominant	61	15.4	0.8
	Yes: Non-dominant	54	13.7	0.02
	No	332	84.0	-
	**Total**	**447**	**113.1**	**-**
Passive flexion of thumb to distal anterior forearm	Yes: Dominant	56	14.2	0.5
	Yes: Non-dominant	47	11.9	0.04
	No	331	83.8	-
	**Total**	**434**	**109.9**	**-**
Passive hyperextension of MP joint of LF	Yes: Dominant	52	13.2	0.8
	Yes: Non-dominant	41	10.4	0.4
	No	339	85.8	-
	**Total**	**432**	**109.4**	**-**
Bend trunk forwards to touch floor with knees in extension	Yes	116	29.4	0.7
	No	279	70.6	-
	**Totals**	**395**	**100**	**-**

MP, metacarpophalangeal; LF, little finger.

**TABLE 3:** Reported thumb hypermobility and association with work-related thumb problems.

Joint	Category	n	%	p-value
IP joint hyperextension (CMC, MP in E): Dominant	Yes, between 0° and 30°	177	**44.8**	0.5
	Yes, > 30°	82	20.8	0.1
	No	136	34.4	-
IP joint hyperextension (CMC, MP in E): Non-dominant	Yes, between 0° and 30°	174	**44.1**	0.8
	Yes, > 30°	79	20.0	**0.02**
	No	142	35.9	-
MP joint hyperextension (CMC joint in E): Dominant	Yes, between 0° and 30°	183	**46.3**	0.2
	Yes, > 30°	54	13.7	0.6
	No	158	40	-
MP joint hyperextension (CMC joint in E): Non-dominant	Yes, between 0° and 30°	179	**45.3**	0.2
	Yes, > 30°	52	13.2	0.6
	No	164	41.5	-
MP joint hyperextension (CMC joint in F): Dominant	Yes, between 0° and 30°	196	**49.6**	0.2
	Yes, > 30°	54	13.7	0.2
	No	145	36.7	-
MP joint hyperextension (CMC joint in F): Non-dominant	Yes, between 0° and 30°	194	**49.1**	0.1
	Yes, > 30°	53	13.4	0.1
	No	148	37.5	-

IP, interphalangeal; CMC, carpometacarpal; MP, metacarpophalangeal; E, extension; F, flexion.

**TABLE 4:** Prevalence of work-related thumb problems in respondents who have thumb hypermobility > 30°.

Category	IP joint hyper E (CMC/MP joints in E): Dominant	%	IP joint hyper E (CMC/MP joints in E): Non-dominant	%	MP joint hyper E (CMC joint in E): Dominant	%	MP joint hyper E (CMC joint in E): Non-dominant	%	MP joint hyper E (CMC joint in F): Dominant	%	MP joint hyper E (CMC joint in F): Non-dominant	%
WRTP	62	75.6	62	78.5	37	68.5	34	65.4	38	70.3	38	71.7
No WRTP	20	24.4	17	21.5	17	31.5	18	34.6	16	29.7	15	28.3
Total	82	100	79	100	54	100	52	100	54	100	53	100

WRTP, work-related thumb problems; IP, interphalangeal; E, extension; CMC, carpometacarpal; MP, metacarpophalangeal; F, flexion.

The factors that were significantly associated with WRTP in all 395 respondents were put in a regression analysis (Table 5). Only hyperextension > 30° of the non-dominant IP joint of the thumb and cervical spine treatment of up to six patients a day remained significantly associated with WRTP. The factors that were not significantly associated with WRTP were all demographic factors, education factors and work areas of the physiotherapists (*p* > 0.05).

**TABLE 5:** Relationship between significant factors and the presence of work-related thumb problems.

Variable	Category	WRTP	%	Odds ratio (OR)	95% Confidence interval	p-value
Cervical spine treatment	1–2 patients	78	30.2	1	-	-
	3–4 patients	125	48.8	1.9	1.2–3.0	0.01
	5–6 patients	40	15.5	2.7	1.3–5.7	0.01
	7–8 patients	9	3.5	1.3	0.4–4.0	0.61
	9–10 patients	0	0	-	-	-
	10–12 patients	0	0	-	-	-
	> 10 patients	1	0	-	-	-
IP hyper-E (CMC/MP joints in E): Non-dominant	No	89	34.5	1	-	-
	Yes (0° – 30°)	107	41.5	1.4	0.6–3.4	0.4
	Yes (> 30°)	62	24.0	4.3	1.01–18.1	0.05
Knee hyper-E > 10°	No	209	81.0	1	-	-
	Dominant	48	18.6	1.2	0.2–6.8	0.8
	Non-dominant	43	16.7	1.9	0.9–4.0	0.1
Thumb to distal forearm	No	209	81.0	1	-	-
	Dominant	41	15.9	1.2	0.3–4.4	0.8
	Non-dominant	37	14.3	1.6	0.7–3.7	0.3

IP, interphalangeal; E, extension; CMC, carpometacarpal; MP, metacarpophalangeal.

Of the 258 physiotherapists who reported WRTP, 254 reported on the timing of the initial incident (Table 6). The majority (40.5%, *n* = 103) of these reported WRTP in their dominant thumbs within 5 years of graduating. Tables 7 and 8 show the prevalence and association of occupational factors with WRTP.

**TABLE 6:** Timing of initial episode of work-related thumb problems.

Timing of initial episode	Dominant thumb	%	Non-dominant thumb	%
Undergraduate	11	4.3	6	2.4
0–5 years after graduating	103	40.5	69	27.2
6–10 years after graduating	51	20.1	43	16.9
10–20 years after graduating	50	19.7	42	16.5
>**&x00A0;**20 years after graduating	19	7.5	18	7.1
Do not know	2	0.8	5	2.0
No problem	18	7.1	71	28.0

*N* = 254.

**TABLE 7:** Prevalence and association of occupational factors with work-related thumb problems.

Work-related factor	n	%	p-value
Soft tissue techniques using thumbs	217	85.4	< 0.001
Joint mobilisation/manipulation techniques	211	83.1	< 0.001
Treating large number of patients a day	159	62.6	< 0.001
Performing same task over and over	156	61.4	< 0.001
Increase in thumb use	150	60.2	< 0.001
Continue to work when thumb is injured	143	56.3	< 0.001
Inadequate training in thumb injury prevention	80	31.5	< 0.001
Working at or near physical limits	44	17.3	< 0.001
Not enough rest breaks	43	16.9	< 0.001
Percussion, vibration, shaking	14	5.5	< 0.001

*N* = 254.

**TABLE 8:** Prevalence and association of spinal mobilising techniques with work-related thumb problems.

Mobilisation technique	Category	n	%	p-value
Central PA pressures	Grade I	8	3.1	0.1
	Grade II	42	16.5	0.1
	Grade III	147	57.9	< 0.001
	Grade IV	112	44.1	< 0.001
Unilateral PA pressures	Grade I	6	2.4	< 0.001
	Grade II	47	18.5	< 0.001
	Grade III	153	60.2	< 0.001
	Grade IV	112	44.1	< 0.001
Transverse glides	Grade I	9	3.5	< 0.001
	Grade II	45	17.7	< 0.001
	Grade III	104	40.9	< 0.001
	Grade IV	82	32.3	< 0.001
Mulligan techniques	Grade I	2	0.01	0.5
	Grade II	29	33.8	< 0.001
	Grade III	66	26	< 0.001
	Grade IV	49	19.3	< 0.001

*N* = 254.

PA, posterior-anterior.

## Discussion

The lifetime prevalence of WRTP for the physiotherapists in the current South African study was 65.3% (*n* = 258). This is similar to the findings of a study in Australia by McMahon *et al.* (2006) which also reported on a national sample of physiotherapists and identified a lifetime prevalence of 65.3% (*n* = 961). Similarly, Barnes *et al.* (2011) reported a lifetime prevalence of wrist and thumb problems of 62.5% (*n* = 55) amongst South African respondents. The lifetime prevalence may have been lower if only thumb problems were reported.

Wajon and Ada (2003) reported a higher lifetime prevalence of WRTP (83%, *n* = 129). The different demographic samples could account for the higher lifetime prevalence. A cross-sectional design including all physiotherapists using manual therapy techniques was used in the current study and in that of McMahon *et al.* (2006), whereas Wajon and Ada (2003) only included physiotherapists who had postgraduate qualifications in manipulative therapy. If only manipulative therapy graduates were included in this study, it would exclude the newly qualified physiotherapists who are more likely to develop WRMSD (Barnes *et al.* 2011; Cromie *et al.* 2000; West & Gardner 2001).

In this study, newly qualified physiotherapists in the age group 20–30 years had a higher prevalence of WRTP (70%, *n* = 84). The reason for the increased prevalence rates in the younger physiotherapists, according to Cromie *et al.* (2000), is the reluctance of younger physiotherapists to ask for help. It could also be linked to the increased workload or inability to stabilise their thumbs during the application of manual therapy techniques (Barnes *et al.* 2011; Buckingham, Das & Trott, 2007; Walsh *et al.* 2011). The repetitive use of their thumbs during manual therapy techniques, especially on large and muscular patients, may pose a challenge which could possibly result in their thumb joints assuming hyperextended positions. Although this study did not find a significant association between WRTP and metacarpophalangeal (MP) joint hypermobility, McMahon *et al.* (2006) found a significant association between WRTP and MP and IP joint hyperextension (*p* < 0.001).

This study showed a significant association between WRTP and non-dominant thumb IP joint hyperextension (*p* = 0.02). Buckingham *et al*. (2007) and Wajon, Ada and Retshauge (2007) advocated that the thumb IP joint be positioned in extension or slight flexion to avoid WRTP, which is supportive of the finding. In contrast, Snodgrass and Rivett (2002) theorised that the lack of hyperextension of the IP joint during the application of posterior-anterior (PA) mobilisation to the spine would result in an increase in WRTP because the base of the thumb is positioned further away from the point of contact when the thumb pad is applying the pressure.

An understanding of the biomechanics of the different thumb joints is important in the prevention of WRTP. This is especially important when the biomechanics of the basal joint of the thumb (carpometacarpal joint) is considered. The osseous configuration of this joint allows for maximum mobility for the intricate functions of the hand; however, there are positions where there is incongruity of the joint surfaces (carpometacarpal joint in retroposition), resulting in decreased stability of the joint (Zancolli, Ziadenberg & Zancolli 1987). For this reason, Atkinson and Maher (2004) questioned physiotherapists’ use of the thumbs in the application of longitudinal pressure during manual therapy techniques. In order to lessen stress to the therapists’ thumb joints, Hu, Hsu and Su (2009) recommended a position in which the thumb is supported by the neighbouring index finger. Jull (2011) describes a modified hand placement for the cervical posterior-anterior glide techniques in which the hands and thumbs are fixed and the force is generated by forearm (elbow) flexion and extension. Walsh *et al.* (2011), Buckingham *et al.* (2007) and Wajon *et al.* (2007) recommended support of the thumbs in the optimal position (thumb in opposition, MP and IP joints in extension or slight flexion) by using taping or splints, especially for physiotherapists who have hypermobility of the thumb joints.

Thistlethwaite's (2005) hypothesised a tendency toward hypermobility as a reason for a statistically significant difference (*p* = 0.005) in race groups; however, the current study found no significant association between WRTP and race. The difference in race demographics could explain this difference. In the current study, 85% of the respondents were white, whilst in Thistlethwaite's (2005) study over 50% of the study population was Indian. She hypothesised that Indian physiotherapists, with a tendency toward hypermobility (Simpson 2006), would increase the tendency for joint instability and the development of WRTP. McMahon *et al.* (2006) reported a higher prevalence of WRTP in male physiotherapists, whereas the current study found a higher prevalence of WRTP in female physiotherapists. These differences could be explained by the difference in gender distribution in each context. In South Africa, physiotherapy is a female-dominated profession. Statistics obtained from the SASP for 2013 indicated that women represented 87.8% of the physiotherapists registered with the society. In this study, women represented 93% of the sample (*n* = 226 of the 243 that responded to the gender questionnaire) whilst in the study by McMahon *et al.* (2006), men represented 22% of the sample. A significant association of WRTP in male physiotherapists working in orthopaedic outpatient departments was also found by McMahon *et al.* (2006), who attributed this to the fact that male physiotherapists are mostly employed in work areas requiring greater use of manual techniques.

The number of patients treated with cervical spine pathology was significantly associated with WRTP in the physiotherapists in this study. Alternatives such as the use of the pisiform are not usually possible in the cervical region because of the contours in this body region, whereas thoracic and lumbar regions lend themselves to the use of the heel of the hand for the stronger grades of movement. McMahon *et al.* (2006) as well as the current study also found a significant association between the presence of WRTP and the hands-on activity of manual therapy, trigger point therapy and massage. A greater percentage of respondents (85.4%, *n* = 217) said that soft tissue techniques aggravated their symptoms when compared to studies by McMahon *et al.* (2006), Wajon and Ada (2003) and Barnes *et al.* (2011) (75%, *n* = 439; 69%, *n* = 106 and 65.5%, *n* = 36 respectively). Specific to the technique of passive accessory spinal joint mobilisation, 80% of the respondents in this study reported aggravation of WRTP. Wajon and Ada (2003) reported the same whilst McMahon *et al.* (2006) reported a slightly lower percentage (75%). Only the current study reported on the grades of the techniques that aggravated WRTP.

A significant association was found between the presence of WRTP and grades III–IV Maitland central posterior-anterior glides, grades II–IV Mulligan techniques as well as all grades of the Maitland unilateral posterior-anterior glides and transverse glides to the spine (*p* < 0.001). An explanation could be that less force is used by the thumbs during the application of the grade I and II techniques compared to the force used in the stronger grades of movement. All Maitland unilateral posterior-anterior glides and transverse glides to the spine, however, were significantly associated with WRTP, perhaps because of the position of the thumb during the application of the technique. The thumb is less likely to be in a position of congruence (carpometacarpal [CMC] in opposition) when performing all grades of the transverse glides, resulting in more stress to the joint surfaces and surrounding soft tissue (Atkinson & Maher 2004; Zancolli *et al.* 1987).

Other occupational factors associated with WRTP in physiotherapists treating musculoskeletal conditions using manual therapy techniques included the high, repetitive workload, treating a large numbers of patients daily, working with a current injury, working in sustained positions for prolonged periods, working in uncomfortable positions, inadequate preventative thumb injury training, inadequate rest periods and working at their physical limits. This was reported by 61% – 80% of the respondents in the current study and other studies reporting on WRTP in physiotherapists (Barnes *et al.* 2011; McMahon *et al.* 2006; Wajon & Ada 2003).

### Limitations of the study

As this study was mainly retrospective in design, the information received was associated with a recall bias and based purely on the respondent physiotherapists’ opinions, interpretation and memory. In addition to this, because of the cross-sectional design of the study, conclusions cannot be drawn regarding the cause or effect of thumb problems ascribed to work-related factors. Internet-based questionnaire bias may influence the validity of the results; Klovning, Sandvik and Hunskaar (2009) reported that a web-based survey reported a higher illness severity and attracted an age-based sample, namely younger respondents. Although the content validity of the questionnaire was addressed by a panel of ‘experts’, the test-retest reliability of the questionnaire was not verified although it should have been. Gender was excluded from the initial questionnaire because of a technical error. The results therefore give a close but not exact representation of the gender ratios for the study. However, they do align with the male to female ratio of the overall number of physiotherapists registered with the SASP.

## Conclusion and recommendations

This study confirms that a high percentage of physiotherapists using manual therapy techniques to treat musculoskeletal conditions are experiencing WRTP. It would seem important that physiotherapists apply the same advice they would give patients who have musculoskeletal disorders. This advice, an important management strategy for WRTP, includes joint protection, job rotation and avoiding sustained postures or repetitive action (Glover 2002).

A longitudinal study which follows newly qualified physiotherapists for at least two years is recommended to investigate a possible cause-effect relationship for WRTP in physiotherapists. Randomised controlled trials that investigate preventative strategies like taping or splints for the physiotherapists’ thumbs are also recommended. If emerging research on this subject is communicated to undergraduate students and practising physiotherapists on an on-going basis, preventative strategies could be implemented. This would prevent physiotherapists from moving to another field of practice or, worse still, leaving the profession as a result of WRTP (McMahon *et al.* 2006; Snodgrass & Rivett 2002; Wagon & Ada 2003).
